# Nascent transcription upon interferon-α2 stimulation on human and rhesus macaque lymphoblastoid cell lines

**DOI:** 10.1186/s13104-023-06465-1

**Published:** 2023-10-26

**Authors:** Daniel Ramirez, Edward B. Chuong, Robin D. Dowell

**Affiliations:** Department of Molecular, Cellular, and Developmental Biology; BioFrontiers Institute, University of Colorado Boulder, Boulder, CO USA

**Keywords:** Homo sapiens, Macaca mulatta, PRO-seq, Type I interferon, Lymphoblastoid cell lines

## Abstract

**Objectives:**

The interferon-triggered innate immune response has been observed to be under strong diversifying selection to counteract the many pathogens hosts have to defend against. In particular, rewiring of gene transcription regulation allows organisms to rapidly acquire new phenotypes by removing and adding genes into the innate immune gene network. Dissecting the molecular processes by which this rewiring takes place, either by changing the DNA regulatory elements or by changing the activity of the regulators across species, is key to better understand this evolutionary process.

**Data description:**

To better comprehend the evolutionary dynamics that have occurred in the initial transcriptional response to interferon in primates, we present Precision Run-On (PRO-seq) datasets made after 1 h of interferon-α2 stimulation on human and rhesus macaque lymphoblastoid cell lines. Further, we tested the difference between using either species’ cognate interferon versus using the other orthologous interferon to account for any potential impacts in the interaction of the orthologous interferons with their cellular membrane receptors. This data provides insights into the regulatory mechanisms that drive species-specific responses to environmental perturbations, such as the one driven by the interactions of pathogens and their hosts.

## Objective

Eukaryotic gene transcription is controlled by promoters and enhancers that are proximal and distal, respectively, to the genes they regulate [1]. When these regulatory elements are actively used by the cell to control gene expression, they are transcribed, typically from both DNA strands originating from a shared RNA polymerase loading locus. These nascently transcribed molecules play diverse roles in the regulation of the genes these regulatory elements are targeting [2, 3].

Regulatory elements have been shown to evolve more rapidly than the genes they regulate [4], which suggest that organisms rely on rewiring gene transcription regulation to quickly acquire new phenotypes. In particular, the cell-intrinsic innate immune system controlled by the interferon (IFN) cytokines has been observed to be one of the most rapidly evolving gene networks [5–7].

IFN molecules are released upon pathogen recognition, and once sensed by cell membrane receptors trigger the formation of transcription factor complexes composed by members of the IRF and STAT protein families, which then regulate the transcription of interferon stimulated genes (ISGs) [8]. However, it has remained an important question in the field how is the rewiring of gene transcription regulation established when evolutionary changes can simultaneously occur both in the regulators themselves, such as mutations in transcription factors, and in changes in the DNA regulatory sequences of the genes these regulators target [9]. Furthermore, biophysical studies demonstrate that varying binding affinities between IFN paralogs to their receptors can ensue distinct cellular responses [10, 11], which may also affect IFN orthologs across species.

Here we address these questions by testing the difference in the transcriptional response when using species-matching compared to non-species matching IFN-α2 treatments on cells derived from human and rhesus macaque, which diverged around 25 mya [12]. These datasets will be of value to researchers who want to investigate the evolutionary changes in the interferon-triggered innate immune transcriptional response in primates.

### Data description

We obtained precision run-on sequencing (PRO-seq) datasets from Epstein-Barr Virus-transformed lymphoblastoid cell lines (LCLs) derived from both a male and a female individual from two primates: human (Homo sapiens, GM12878 and HG03077 from the Coriell Institute for Medical Research) and rhesus macaque (Macaca mulatta, Mm 150 − 99 and Mm 290 − 96 generously shared by Yoav Gilad from the University of Chicago) (see Data set 1 in Table 1). Each primate cell line was treated for 1 h with either the vehicle bovine serum albumin (BSA), with their cognate species-matching IFN-α2, or with the other primate’s orthologous IFN-α2. Libraries were prepared as described in [13] with minor modifications, see methods file (Data file 3) for full details. Libraries were sequenced on an Illumina NextSeq 500 using single-end sequencing, on which an average of 36 million reads per library were obtained. Raw reads (available at NCBI GEO under accession number GSE214304) were assessed for quality (see data file Data file 1) and mapped to the respective species genomes (hg38 for Homo sapiens and rheMac10 for Macaca mulatta) (see Data file 3 for full details).

Briefly, we counted reads over gene bodies using featureCounts and used DESeq2 [14] and known ISGs to validate that the LCLs responded as expected to the IFN-α2 treatments. Additionally, we assessed the consistent simulation of key transcription factors upon IFN-α2 by first detecting bidirectionally transcribed loci using Tfit [15] and dREG [16]. These bidirectionally transcribed loci, roughly corresponding to enhancers and promoters, are then fed to TFEA to identify transcription factors responding to IFN-α2 treatment [17] (see Data file 3 for full details).

The human IFN-α2 and rhesus IFN-α2-treated LCLs display a typical type I interferon stimulation transcriptional response compared to the BSA control datasets, and similar to published nascent transcription response to IFN-γ on mouse embryonic fibroblasts (MEFs) [18] (see Data file 2, Data file 4). The human IFN-α2-treated datasets, however, show a greater interferon stimulation magnitude than the rhesus IFN-α2-treated datasets, regardless of the primate LCL used.

**Table 1:** Overview of data files/data sets

Label	Name of data file/data set	File types (file extension)	Data repository and identifier (DOI or accession number)
*Data file 1*	*Quality control assessment of data sets sequencing.*	*MultiQC file (.html)*	*Figshare* (10.6084/m9.figshare.21253245.v1) [21]
*Data file 2*	*Assessment of interferon stimulation of data sets.*	*PDF file (.pdf)*	*Figshare* (10.6084/m9.figshare.21287637.v1) [22]
*Data file 3*	*Detailed methods file.*	*PDF file (.pdf)*	*Figshare* (10.6084/m9.figshare.21287652.v1) [23]
*Data file 4*	*DESeq2 results tables*	*Tab separated files (.tsv)*	*Figshare* (10.6084/m9.figshare.23971908) [24]
*Data set 1*	*PRO-seq datasets of Homo sapiens and Macaca mulatta LCLs treated with either BSA, human IFN-*α*2, or rhesus IFN-*α*2.*	*fastq (.fastq.gz)*	*NCBI GEO* (https://identifiers.org/geo:GSE214304) [25]

### Limitations

The human IFN-α2 protein was obtained from Proteintech Cat. no. HZ-1066, whereas the rhesus macaque IFN-α2 was obtained from PBL Assay Science Ca. no. 16105-1. Each manufacturer tested their purified protein activities using different assays, with the human IFN-α2 protein purification having been tested with a “dose-dependent cytotoxicity of the human TF-1 cell line (human erythroleukemic indicator cell line)” [19], and the rhesus IFN-α2 protein purification with a “cytopathic inhibition assay on Bovine (MDBK) kidney cells with vesicular stomatitis [virus] (VSV)” [20]. Discrepancies in the bioactivity assay details may have resulted in unequal magnitude of IFN-dependent transcriptional responses even when using 100 units/mL for both the human and rhesus IFN-α2 protein treatments. To this end, we observe that both cell lines responded more strongly to the human IFN-α2, as observed by the number of differentially transcribed genes and by the magnitude of the ISGs fold-change.

While we assayed two distinct cell lines per species, one female and one male, each biological sex was only assayed once.

## Data Availability

The data described in this Data note can be freely and openly accessed on NCBI under GEO accession number GSE214304. Please see Table 1 and references *Data file 1, 2, 3, & 4* and *Data set 1* for details and links to the data.

## Abbreviations

PRO-seq: Precision Run-On followed by sequencing
IFN-α2: Interferon alpha2
BSA: Bovine Serum Albumin
PBS: Phosphate-Buffered Saline
ISGs: Interferon Stimulated Genes
LCL: Lymphoblastoid Cell Line
MEFs: Mouse Embryonic Fibroblasts
